# Cholangioscopy-assisted extraction of choledocholithiasis through novel papillary support: the protective effect on the sphincter of Oddi

**DOI:** 10.1055/a-2316-0924

**Published:** 2024-06-18

**Authors:** Zixin Wang, Enqiang Linghu, Longsong Li, Wengang Zhang, Nan Ru, Bo Zhang, Ningli Chai

**Affiliations:** 1 Department of Gastroenterology, The First Medical Center of Chinese People’s Liberation Army General Hospital, Beijing, China

A 78-year-old man with choledocholithiasis underwent cholangioscopy-assisted extraction for multiple stones with a maximum diameter of 10 mm ( Fig. 1 ). Considering the potential loss of sphincter of Oddi (SO) function associated with endoscopic sphincterotomy and endoscopic papillary balloon dilation, we used novel papillary support to provide an adequate exit for the extraction of the stones 1 2 . The papillary support (12 mm in diameter, 25 mm in length) is a metal-covered membrane stent with a unique single dumbbell-style design on the papillary side, serving to protect the support from entering the common bile duct (CBD) during stone removal procedures 3 .

**Fig. 1:**
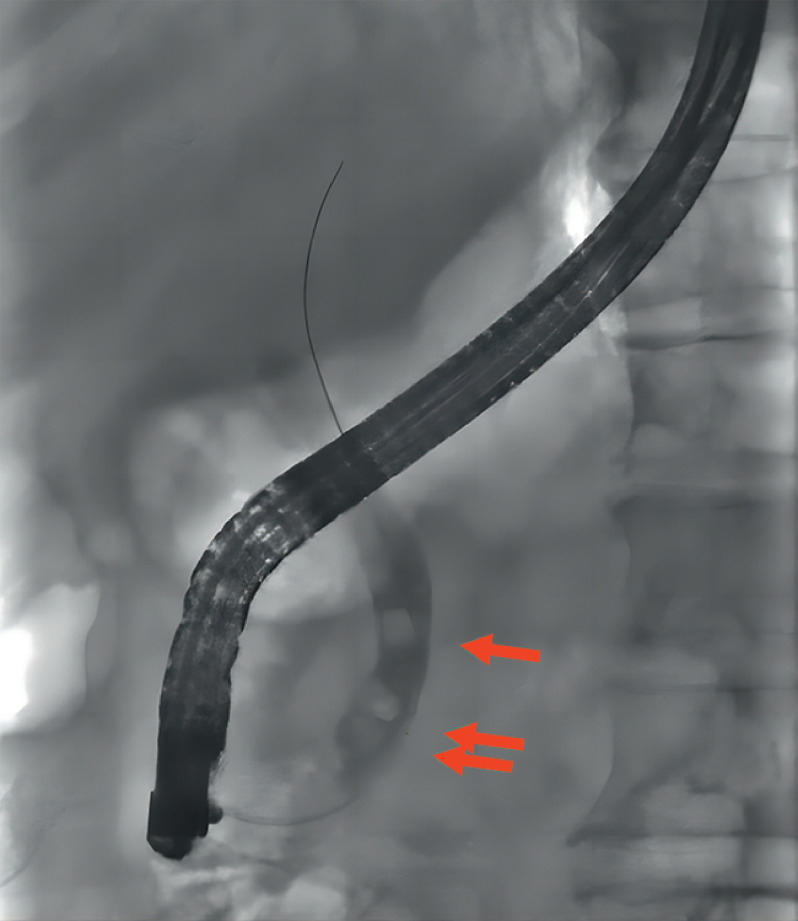
Endoscopic retrograde cholangiopancreatography fluoroscopic image showing multiple stones (maximum diameter of 10 mm, arrows) in the lower common bile duct (12 mm).

After biliary intubation, the papillary support was inserted into the distal CBD to facilitate stone removal. The cholangioscopy (eyeMax, 9F; Micro-Tech, Nanjing, China) was inserted into the CBD through the papillary support ( Fig. 2 ). Then the stones were found directly under cholangioscopy. Stone extraction was accomplished using a basket ( Fig. 3 ). Subsequently, the papillary support was removed, and a biliary plastic stent (7F, 6 cm) was placed in the CBD ( Video 1 ). Notably, no postoperative pancreatitis, bleeding, or other adverse events were observed.

**Fig. 2:**
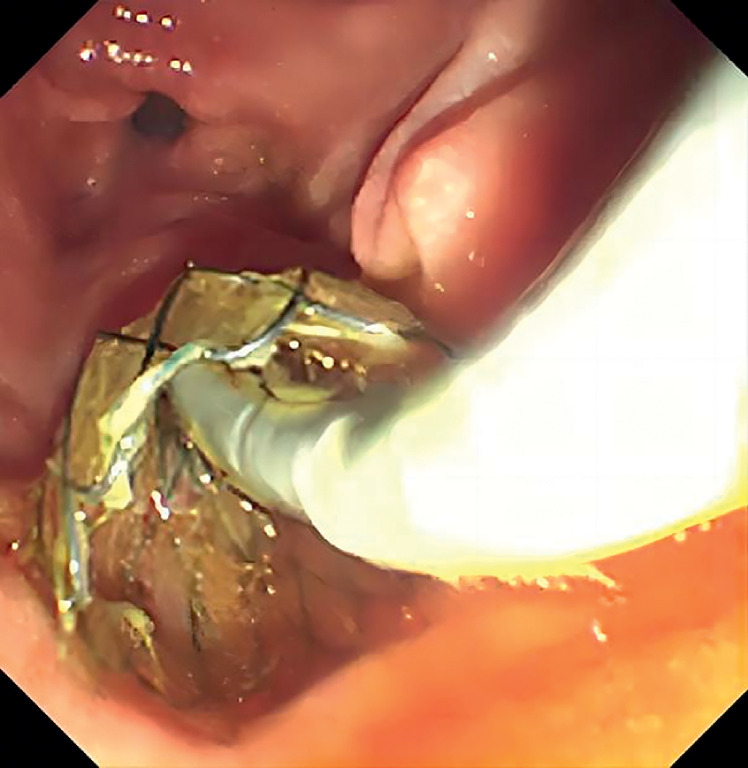
The papillary support was inserted into the distal common bile duct.

**Fig. 3:**
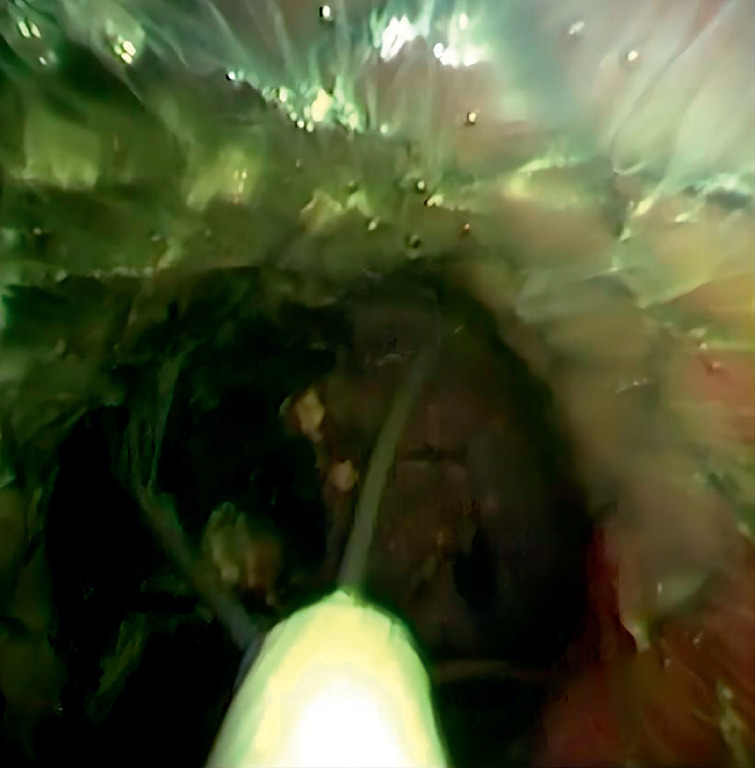
Stones were extracted with a basket under the choledochoscopy through papillary support.

Cholangioscopy-assisted extraction was performed through novel papillary support for choledocholithiasis.Video 1

In this case, we conducted sphincter of Oddi manometry (SOM) both before and after the placement of the papillary support ( Fig. 4 ). The patient exhibited normal SO function before the procedure. However, after removing the papillary support, there was an immediate decline in CBD pressure, SO basal pressure, amplitude, and frequency of contractions ( Fig. 5 ). One week later, the stent was spontaneously removed, and we conducted SOM again to evaluate SO function. The SO basal pressure, amplitude, and frequency of contractions had recovered to normal range ( Fig. 5 , Video 1 ). This encouraging outcome introduces a new method to preserve SO function. Further investigation is necessary to validate the safety and effectiveness of this technique.

**Fig. 4:**
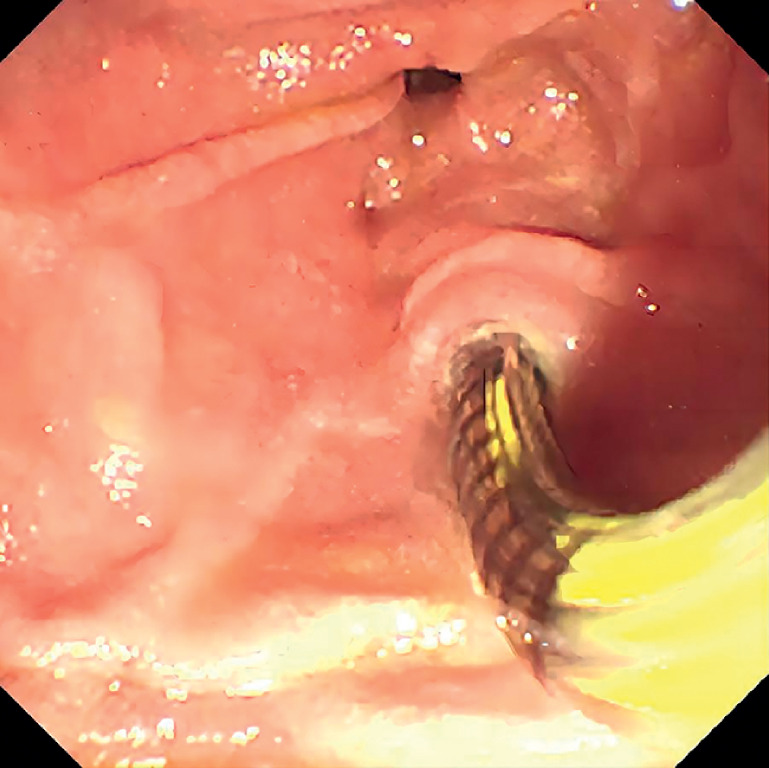
Sphincter of Oddi manometry was performed.

**Fig. 5:**
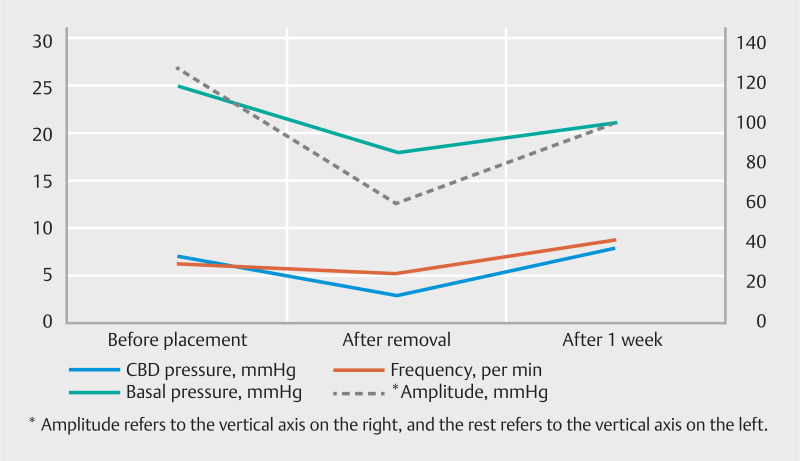
Sphincter of Oddi pressure before and after the placement of the papillary support, as well as after 1 week.

Endoscopy_UCTN_Code_TTT_1AR_2AH

## Notes

Endoscopy E-Videos https://eref.thieme.de/e-videos
*E-Videos* is an open access online section of the journal *Endoscopy* , reporting on interesting cases and new techniques in gastroenterological endoscopy. All papers include a high-quality video and are published with a Creative Commons CC-BY license. Endoscopy E-Videos qualify for HINARI discounts and waivers and eligibility is automatically checked during the submission process. We grant 100% waivers to articles whose corresponding authors are based in Group A countries and 50% waivers to those who are based in Group B countries as classified by Research4Life (see: https://www.research4life.org/access/eligibility/ ). This section has its own submission website at https://mc.manuscriptcentral.com/e-videos .
